# Beyond traditional biomarkers: do serum cystatin C and urinary cystatin B improve diagnostic precision in feline and canine renal disease? A systematic review and meta-analysis

**DOI:** 10.3389/fvets.2026.1844210

**Published:** 2026-07-03

**Authors:** Brisa Miranda Andrade Santos, Beatriz Aline Migotto, Sofia Borin Crivellenti, Caio Santos Pennacchi, Leandro Zuccoloto Crivellenti

**Affiliations:** Graduate Program in Veterinary Sciences (PPGCVET), School of Veterinary Medicine (FAMEV), Federal University of Uberlândia (UFU), Uberlândia, Minas Gerais, Brazil

**Keywords:** glomerular filtration rate, meta-analysis, nephrology, tubular injury, veterinary medicine

## Abstract

Chronic kidney disease (CKD) is the most common nephropathy in cats and dogs, particularly in elderly animals, whereas acute kidney injury (AKI) represents an abrupt-onset condition associated with high morbidity and mortality. Serum creatinine and urea are widely used for renal function assessment; however, they exhibit low sensitivity for the early detection of renal dysfunction. In this context, alternative biomarkers, such as serum cystatin C (sCysC) and urinary cystatin B (uCysB), have been investigated as potential diagnostic tools. The present study conducted a meta-analysis, following PRISMA guidelines, to evaluate the performance of these biomarkers in cats with CKD and dogs with AKI. Three studies on sCysC and two on uCysB were included, comparing diseased cats and dogs with healthy controls, only one article on uCysB in cats met the inclusion and exclusion criteria and was analyzed qualitatively. The meta-analysis demonstrated a consistent increase in sCysC in cats with CKD, with a large effect size, indicating moderate and stable discriminatory ability. Conversely, uCysB showed a marked elevation in dogs with AKI, with effect sizes exceeding thresholds for strong clinical relevance, suggesting a strong association with tubular injury. However, high heterogeneity was observed among uCysB studies, along with relevant methodological limitations, including predominant comparisons with healthy animals. It is concluded that sCysC has limited utility in current clinical practice, with no proven superiority over creatinine or symmetric dimethylarginine (SDMA), whereas uCysB emerges as a promising biomarker for AKI, although its clinical application still depends on more robust prospective studies.

## 1 Introduction

CKD is the most prevalent nephropathy in dogs and cats, with an estimated prevalence ranging from 1.6 to 20% in the general population and affecting up to 35–50% of cats and dogs over 15 years of age (1, 2). It is a progressive and irreversible condition characterized by the gradual loss of renal function, with a significant negative impact on the quality of life and longevity of affected animals. In this context, the early detection of renal dysfunction is crucial to slowing disease progression, instituting appropriate therapeutic interventions, and improving clinical prognosis (3, 4).

In contrast to the insidious course of CKD, AKI is defined as an abrupt reduction in renal function, which can range from minor increases in serum creatinine to complete loss of renal function, and is associated with a significant prognostic impact in hospitalized and critically ill patients (5). The incidence of AKI has increased alongside the complexity of hospital care, being associated with a higher risk of progression to CKD and increased mortality (5, 6).

Glomerular filtration rate (GFR) is considered the gold standard for assessing renal function, as it directly reflects the kidneys’ filtration capacity. However, direct measurement of GFR in animals is laborious, time-consuming, and not readily applicable in clinical practice, making the use of indirect markers indispensable (7). Currently, serum creatinine and urea remain the most widely used biomarkers, but they have significant limitations: both increase only after a loss of more than 75% of functional renal mass and are influenced by extra-renal factors such as dietary intake, drug use, and variations in muscle mass, which are particularly relevant (4). These limitations justify the search for earlier and more reliable biomarkers of renal dysfunction in cats (60).

In this context, CysC emerged as a potentially promising biomarker of renal function. It is a low-molecular-weight protein (approximately 13 kDa), produced at a constant rate by all nucleated cells, freely filtered by the glomeruli, and almost completely reabsorbed and catabolized in the proximal tubules, with no significant tubular secretion or extra-renal elimination (8, 9). These properties theoretically make CysC an ideal endogenous biomarker of GFR (10–12). In humans and dogs, sCysC has demonstrated potentially greater sensitivity than creatinine for the early detection of GFR reduction and for identifying AKI before serum creatinine elevation. Furthermore, detectable urinary cystatin C has been associated with tubulointerstitial injury, reflecting tubular damage (13, 41).

Despite this biotechnological potential, the clinical utility of CysC in cats remains controversial. Previous studies show inconsistent results, with variable correlations between this marker, GFR, and serum creatinine (14, 63). This limitation is compounded by the absence of widely validated commercial assays for the species, restricting its application in routine diagnostics (15).

On the other hand, SDMA, a byproduct of protein methylation eliminated predominantly via renal route, has become established as a sensitive marker of GFR reduction (16). SDMA concentrations are elevated in both AKI and CKD, correlating strongly with serum creatinine in acute conditions (17). However, SDMA lacks specificity regarding the nature of the injury, being unable to distinguish tubular damage from isolated functional loss (16, 17).

More recently, uCysB has emerged as a promising biomarker of AKI in dogs and cats, with higher values being related to worse clinical outcomes (18–20). These findings contrast with the still limited evidence for CysC and suggest that uCysB may represent a more robust marker of acute tubular injury in this species.

Given this context, the need to differentiate biomarkers aimed at assessing glomerular function from those directed at detecting structural renal damage becomes evident. Thus, the present study aims to critically address the role of CysC in feline CKD and uCysB in AKI in dogs, discussing their clinical applicability, limitations, and potential contribution to the early diagnosis, staging, and prognosis of kidney diseases in this species. uCysB meta-analysis was performed in dogs, while feline evidence was included only qualitatively due to the identification of a single eligible feline study.

## 2 Materials and methods

### 2.1 Study design and reporting guidelines

This study was conducted as a systematic review with meta-analysis, in accordance with the recommendations of the Preferred Reporting Items for Systematic Reviews and Meta-Analyses (PRISMA, 2020). The research questions and eligibility criteria were structured using the PECO strategy (Population, Exposure, Comparison, Outcome).

### 2.2 Research questions and PECO strategy

For CysC, the PECO strategy was defined as follows: the population (P) consisted of domestic cats; the exposure (E) corresponded to the presence of CKD; the comparison group (C) included clinically healthy cats; and the outcome (O) was the serum concentration of CysC. Based on this design, the following research question was formulated: “Do cats with chronic kidney disease have higher serum CysC concentrations compared to healthy cats?”

For CysB, the PECO strategy was initially designed in a similar manner, however considering dogs the population (P), AKI as the exposure (E), clinically healthy dogs as the comparison (C), uCysB concentration as the outcome (O). The corresponding research question was: “Do dogs with acute kidney injury have higher uCysB concentrations compared to healthy dogs?”

### 2.3 Information sources and search strategy

A systematic search was performed in the PubMed database, selected for its comprehensiveness and relevance in the biomedical field. For sCysC, the search strategy used the following combination of descriptors and Boolean operators:

(feline OR cats) AND (cystatin C) AND (chronic kidney disease OR CKD).

For uCysB, the following search strategy was employed:

(cystatin B OR uCysB) AND (acute kidney injury) AND (dogs OR canine).

(cystatin B OR uCysB) AND (acute kidney injury) AND (cats OR feline).

**Figure 1:**
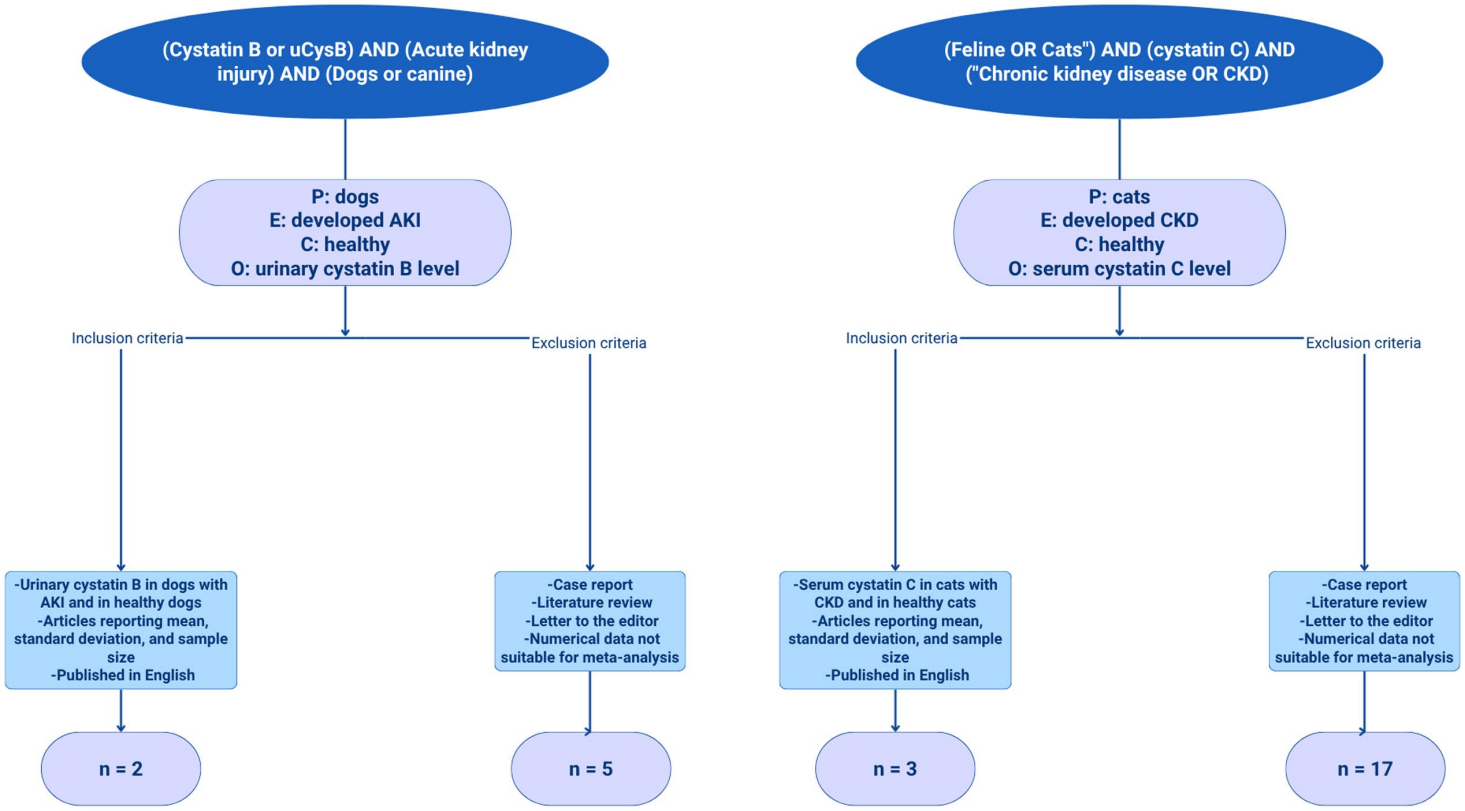
Flowchart of the identification, screening, eligibility, and inclusion process of studies used in the meta-analysis, according to the adopted search strategy.

No restrictions regarding the year of publication were applied. Only studies published in the English language were considered. The search yielded 20 records for sCysC and 7 records for uCysB. The complete study selection process is presented in the flowchart (Figure 1). Only one study evaluating uCysB in cats met the predefined inclusion and exclusion criteria of this systematic review. Although this study fulfilled the qualitative eligibility requirements.

### 2.4 Eligibility criteria

Studies were included if they simultaneously met the following criteria:

1. Original observational studies;
2. Evaluation of sCysC in cats with CKD and AKI or uCysB in dogs with AKI;
3. Presence of a control group composed of healthy cats;
4. Provision of adequate quantitative data for meta-analysis (mean, standard deviation, and sample size);
5. Publication in the English language.

Case reports, narrative or systematic reviews, editorials, letters to the editor, experimental studies not involving CKD or AKI, and studies that did not present numerical data compatible with statistical analysis were excluded. Based on these criteria, three studies were included for sCysC and two studies for uCysB, with 17 and 5 studies being excluded.

### 2.5 Study selection process

Study screening was performed independently by two reviewers, initially through the reading of titles and abstracts. Potentially eligible articles were evaluated in full text to confirm inclusion criteria. Disagreements between reviewers were resolved by consensus.

### 2.6 Data extraction

Data extraction was conducted independently by two reviewers using a standardized electronic spreadsheet. Data regarding study characteristics, sample size, means, standard deviations, and confidence intervals of sCysC and uCysB concentrations in the diseased and control groups were recorded. The extracted data are presented in Tables 1, 2.

**Table 1:** Articles included in the review, according to the eligibility criteria, presenting the country of origin and the methodology employed for serum cystatin C measurement in cats with chronic kidney disease and healthy controls, as well as the main results obtained.

Study	Country	Methodology	Results
Ghys et al. (14)	Belgium	Nephelometric assay	Cats with CKD had significantly higher serum sCysC concentrations compared to healthy controls.
Ghys et al. (15)	Belgium	Nephelometric assay	sCysC concentrations were, on average, significantly higher in cats with CKD than in healthy controls.
Williams et al. (63)	United Kingdom	Particle-enhanced turbidimetric assay	No significant difference in sCysC concentrations was observed when comparing cats with CKD to healthy control cats.

**Table 2:** Articles included in the review, according to the eligibility criteria, presenting the country of origin and the methodology employed for uCysB measurement in dogs with AKI and healthy controls, as well as the main results obtained.

Study	Country	Methodology	Results
Gordin et al. (19)	United States	sandwich ELISA	Healthy dogs showed undetectable or low uCysB levels, while cats with acute kidney injury exhibited markedly elevated urinary concentrations.
Gordin et al. (26)	United States	sandwich ELISA	uCysB concentration differed significantly between groups, being higher in the AKI group compared to controls.
Chen et al. (18)	Israel	sandwich ELISA	uCysB progressively increased in cats with AKI.

Only one article on uCysB in cats met the inclusion and exclusion criteria and was therefore analyzed qualitatively.

### 2.7 Risk of bias assessment

The risk of bias of the included studies was assessed independently by two reviewers using an appropriate tool for observational studies, such as the Newcastle–Ottawa Scale. Any disagreements were resolved by consensus.

### 2.8 Statistical analysis

Statistical analyses were performed using RStudio software. Differences in biomarker concentrations between the CKD vs. control groups for sCysC, and AKI vs. control groups for uCysB were evaluated using meta-analysis under a random-effects model, considering the clinical and methodological heterogeneity among the studies.

Effect sizes were estimated using Cohen’s d and Glass’s delta, with respective 95% confidence intervals. The pooled standard deviation and statistical power based on Glass’s delta were also calculated. Heterogeneity among studies was assessed using Cochran’s Q test and quantified by the I^2^ index, which expresses the proportion of total variability attributed to heterogeneity among studies rather than chance. As this study involved a secondary synthesis of previously published data, it did not involve direct manipulation of animals and, therefore, did not require submission to an animal research ethics committee.

## 3 Results

### 3.1 Difference in means between groups

The comparative meta-analysis included two studies that evaluated the urinary concentration of uCysB in dogs with AKI and three studies that investigated the serum concentration of sCysC CKD in cats, all reporting means, standard deviations, and sample sizes adequate for quantitative synthesis. In both analyses, the initial estimation was conducted using the weighted mean difference, employing a fixed-effect model. The statistical weights assigned to the studies were calculated based on the inverse of the variance, incorporating the standard deviations and sample sizes of the diseased and control groups.

In the analysis of uCysB, the pooled weighted mean difference was 317.65, indicating markedly higher urinary concentrations in dogs with AKI compared to healthy controls. However, the forest plot demonstrated considerable dispersion among studies, with lack of vertical alignment suggesting low homogeneity. Notably, the study presenting the largest difference in means contributed the least weight due to its higher standard deviation and smaller sample size.

**Figure 2:**
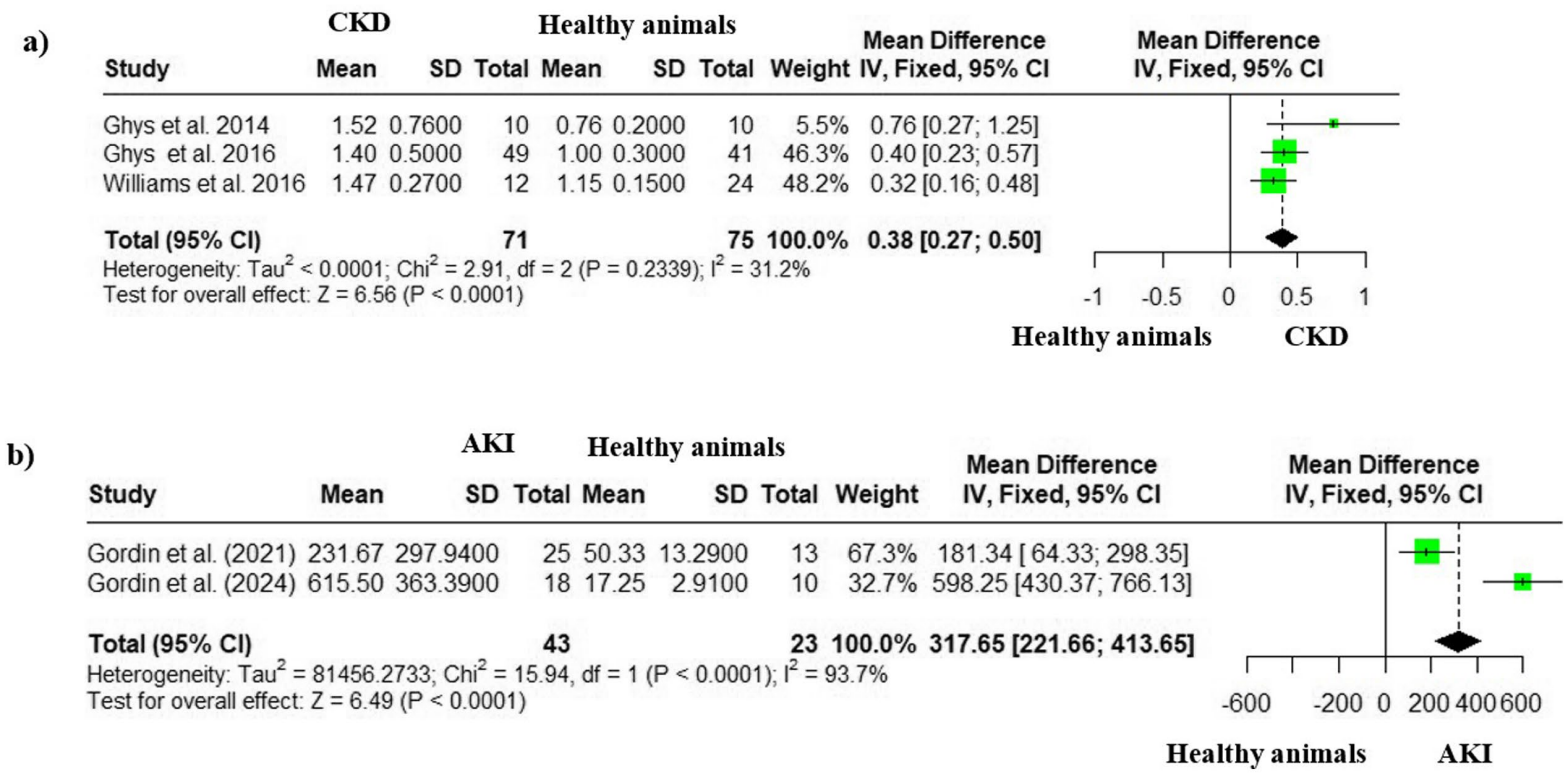
Forest plot of sCysC in cats with CKD and uCysB in dogs with AKI compared to healthy controls. Both biomarkers showed higher levels in diseased animals, with pooled effects of 0.38 (sCysC) and 317.65 (uCysB), indicating clear separation between healthy and diseased groups. Clinically, these results support the discriminatory potential of both biomarkers. **(a)** sCysC; **(b)** uCysB.

Similarly, the meta-analysis of sCysC demonstrated a consistent increase in serum levels in cats with CKD compared to controls. The overall weighted mean difference was 0.38, also represented by the central diamond in the forest plot. Notably, the study that presented the largest absolute difference in means contributed the least statistical weight (5.5%), due to its reduced sample size and high standard deviation, evidencing greater internal variability. As observed for uCysB, studies with greater variability presented wider confidence intervals, suggesting heterogeneity among the individual results (Figure 2).

### 3.2 Heterogeneity and effect models

Heterogeneity among the studies was quantified using Higgins’ I^2^ statistic for both biomarkers, revealing distinct patterns of variability among the included works. In the meta-analysis of uCysB, high heterogeneity was observed (I^2^ = 93.7%), indicating that most of the variability among the results cannot be attributed to sampling error, but likely to uncontrolled factors, such as differences in age, breed, sex, laboratory methods employed, and clinical stages of AKI.

Given this scenario of high heterogeneity, the application of a random-effects model was necessary, aiming to incorporate the variability between studies and provide a more conservative and generalizable estimate. The adoption of this model resulted in a more balanced redistribution of statistical weights, accompanied by an increase in the width of the confidence intervals of the overall estimate, graphically represented by a wider diamond in the forest plot. In this context, the pooled estimate approached the null line (zero), suggesting that, under conditions of high heterogeneity, the behavior of uCysB may vary substantially across populations, and may approximate between control and AKI groups depending on the clinical and demographic characteristics evaluated.

In contrast, the meta-analysis of sCysC presented a distinct pattern. Under the fixed-effect model, partial homogeneity was observed among the studies, with moderate overall heterogeneity (I^2^ = 31.2%). Nevertheless, the complementary application of a random-effects model was chosen, aiming to incorporate residual variability among the studies and produce a more conservative and biologically plausible estimate.

**Figure 3:**
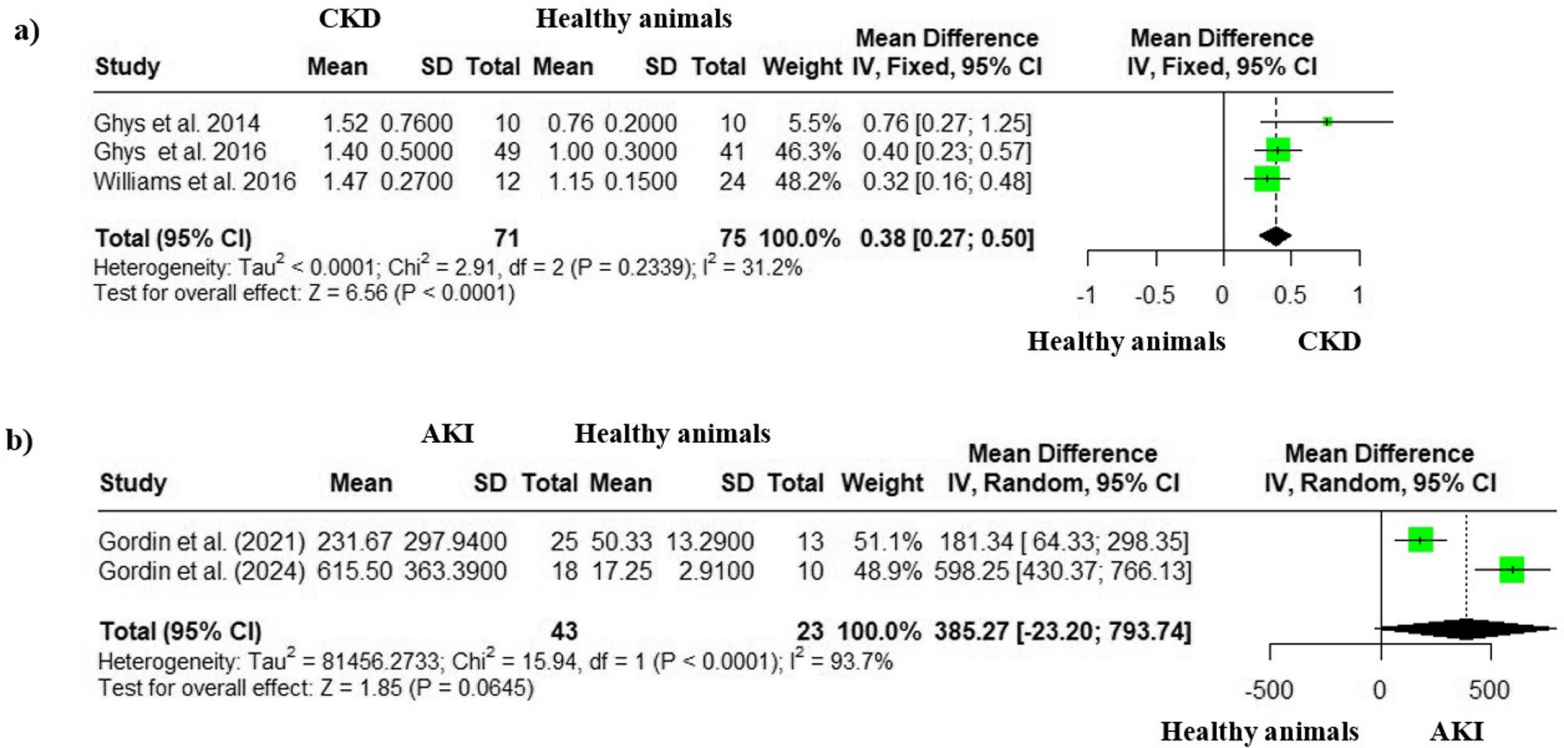
Heterogeneity analysis of sCysC in cats with CKD and uCysB in dogs with AKI. Heterogeneity was assessed using Higgins’ I^2^ statistic, showing high variability for uCysB (I^2^ = 93.7%) and moderate heterogeneity for sCysC (I^2^ = 31.2%).

The application of the random-effects model for sCysC resulted in a reduction in the discrepancy between the weights assigned to the studies and a widening of the confidence intervals of the pooled estimate (Figure 3).

### 3.3 Standardized effect sizes and clinical relevance

To enable comparisons independent of the measurement scale and to assess the clinical relevance of the biomarkers, the meta-analysis results were converted into standardized effect sizes. The effect size expresses the magnitude of the difference between groups and is classified as small, moderate, or large, with at least a moderate to large effect size being expected for diagnostic biomarkers.

**Figure 4:**
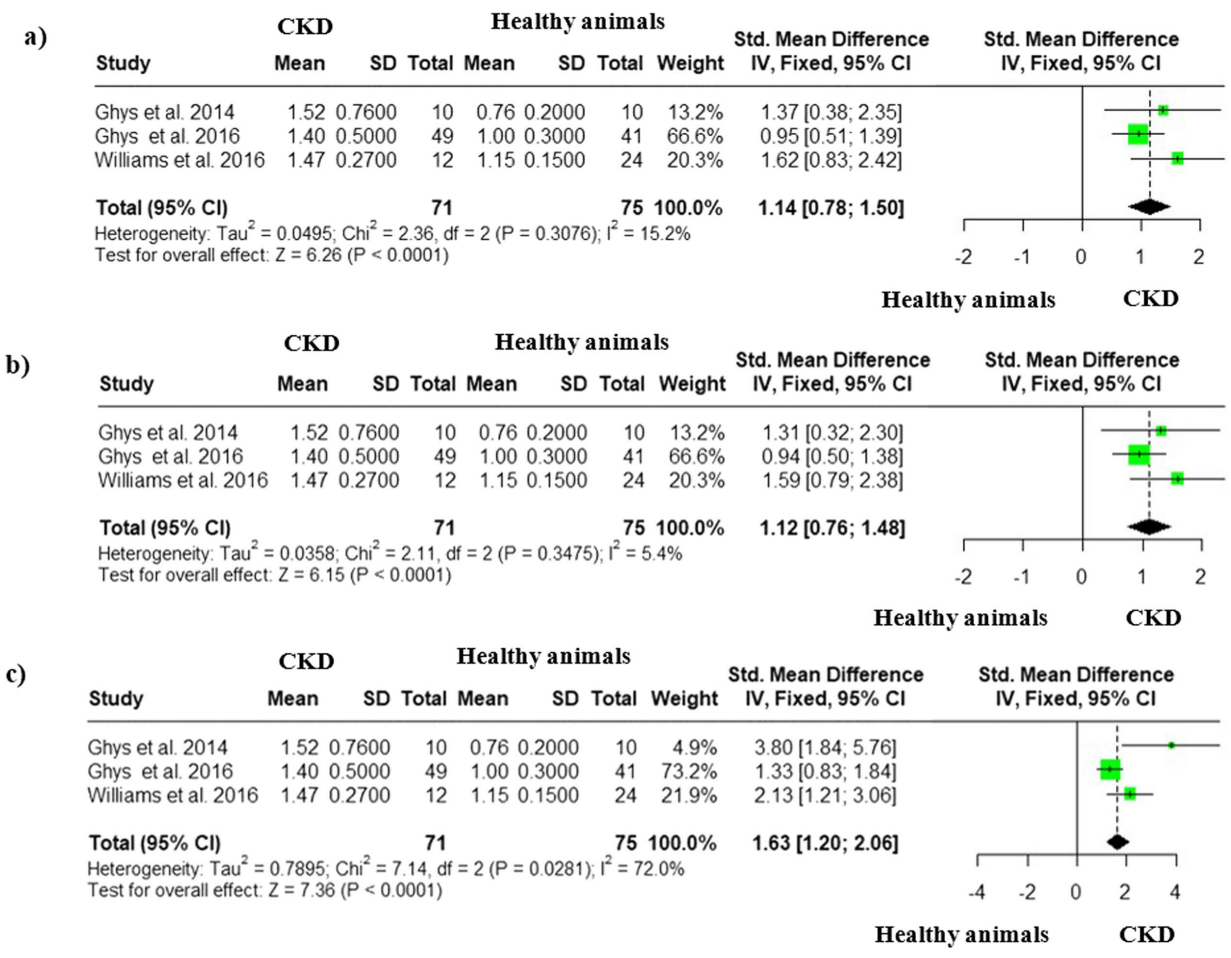
Standardized effect size estimates for sCysC in cats with CKD. sCysC showed consistently large effect sizes across all measures, indicating a strong separation between cats with CKD and healthy controls. Clinically, these findings support the robust discriminatory capacity of sCysC at the population level. **(a)** Cohen’s d for sCysC; **(b)** Hedges’ g for sCysC; **(c)** Glass’s *Δ* for sCysC.

**Figure 5:**
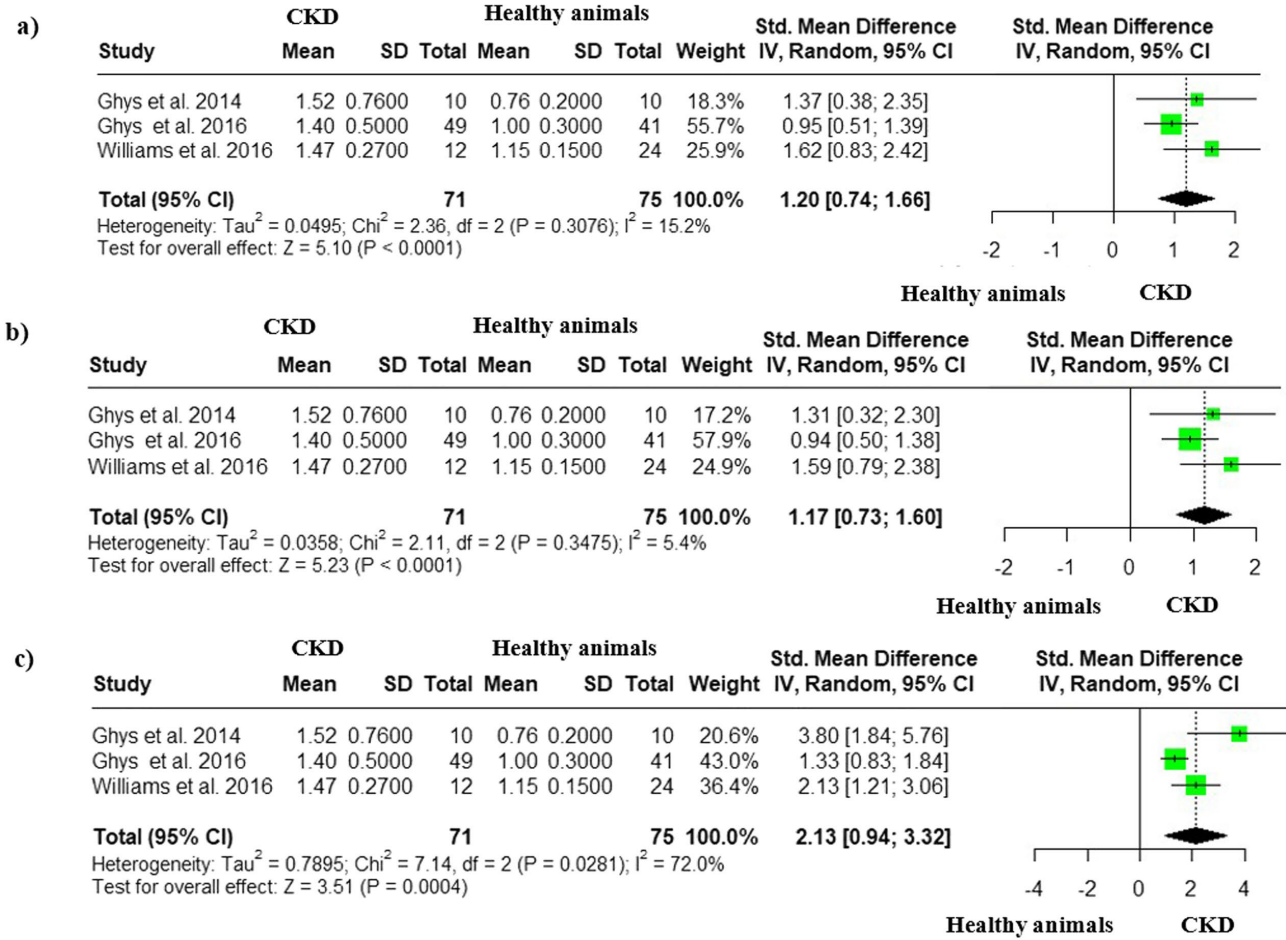
Standardized effect sizes of sCysC in cats with CKD under the random-effects model. Consistently large effect sizes were observed across all metrics, supporting robust discrimination between cats with CKD and healthy controls. Clinically, these findings reinforce the strong group-level discriminatory capacity of sCysC, while accounting for residual between-study variability. **(a)** Cohen’s d for sCysC; **(b)** Hedges’ g for sCysC; **(c)** Glass’s Δ for sCysC.

In the analysis of CysC as a biomarker of CKD, large and consistent magnitudes were observed across different effect size metrics. Cohen’s d indicated a large effect size (d = 1.14), while Hedges’ g, adjusted for small or unbalanced samples, presented a similar value (g = 1.12), confirming the robustness of the observed effect. The application of Glass’s Delta (*Δ*), which exclusively uses the standard deviation of the control group as the denominator, resulted in the largest effect size (Δ = 1.63), suggesting high diagnostic efficiency of sCysC (Figure 4). These findings remained consistent under both the fixed-effect and random-effects models, although, in the latter, a widening of confidence intervals was observed, reflecting greater expected variability in heterogeneous populations. It is important to note that none of the estimates crossed the null line, reinforcing the consistency of sCysC as a biomarker, regardless of factors such as age, sex, or breed (Figures 4, 5).

**Figure 6:**
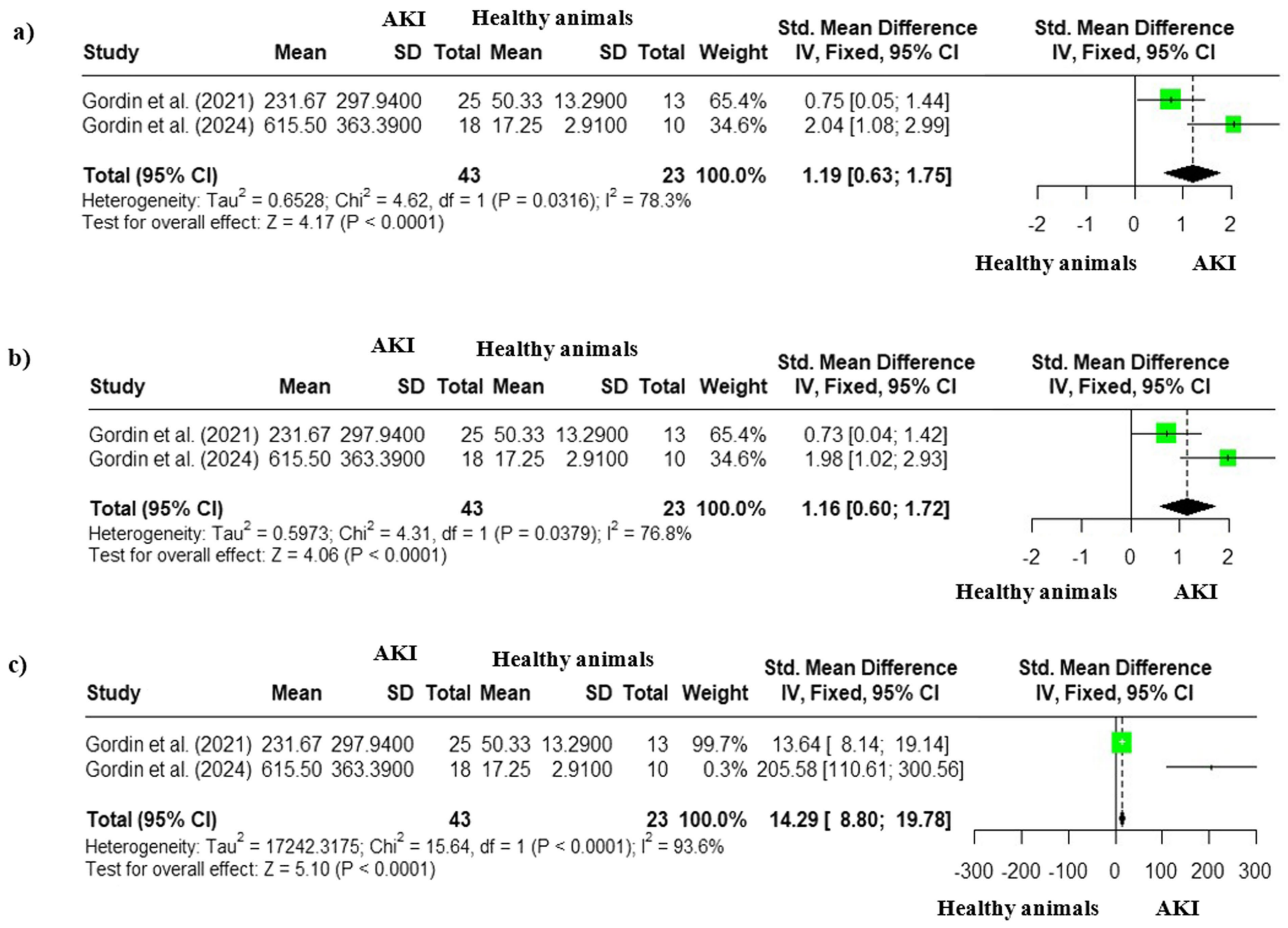
Standardized effect size estimates for uCysB in dogs with AKI. uCysB demonstrated consistently large to very large effect sizes across all measures, indicating strong discrimination between dogs with AKI and healthy controls. Clinically, these findings support the potential value of uCysB as a biomarker of active tubular injury, although further studies are needed to establish clinically applicable diagnostic and prognostic thresholds. **(a)** Cohen’s d for uCysB; **(b)** Hedges’ g for uCysB; **(c)** Glass’s Δ for uCysB.

For uCysB as a biomarker of AKI, conversion to standardized effect sizes revealed exceeding thresholds for strong clinical relevance. Cohen’s d demonstrated a very large effect size (d = 1.19), consistent with high discriminatory capacity between cats with AKI and healthy dogs. Considering the small number of studies and possible sample imbalances, the effect was adjusted using Hedges’ g, which maintained an effect size classified as large (g = 1.16), although with a lower magnitude than initially estimated. Subsequently, the calculation of Glass’s Delta evidenced a substantial increase in effect size (*Δ* = 14.29), indicating impressive performance of uCysB in distinguishing between groups, especially when evaluated in relation to the variability of the control group (Figure 6).

**Figure 7:**
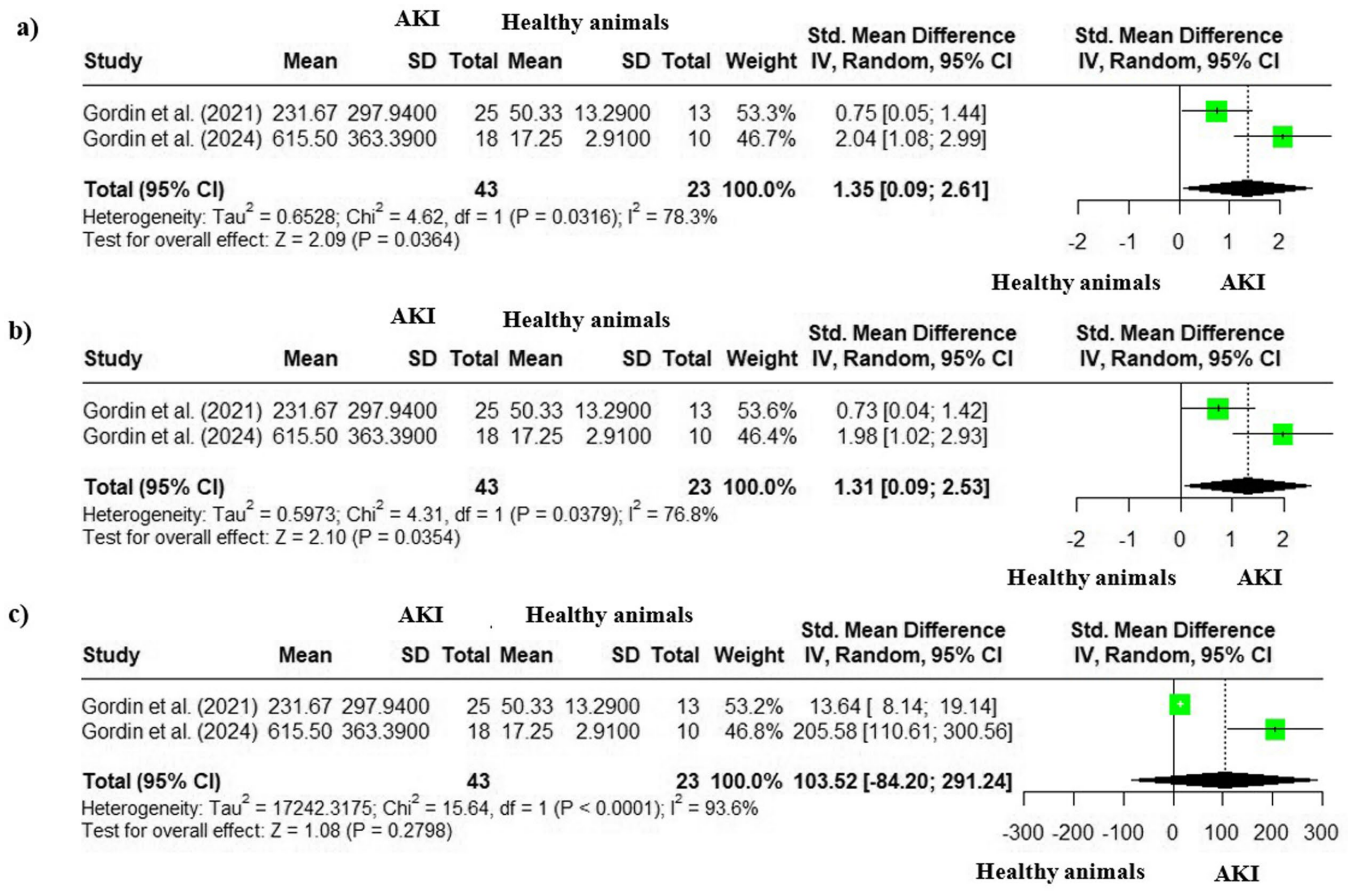
Standardized effect sizes of uCysB in dogs with AKI under the random-effects model. Very large effect sizes were observed across all metrics, indicating strong separation between dogs with AKI and healthy controls. However, wider confidence intervals were observed compared with the fixed-effect model, reflecting greater between-study heterogeneity. Clinically, these findings support the potential value of uCysB as a biomarker of tubular injury while highlighting the influence of population variability on effect estimates. **(a)** Cohen’s d for uCysB; **(b)** Hedges’ g for uCysB; **(c)** Glass’s Δ for uCysB.

The introduction of the random-effects model led to additional adjustments in effect sizes for uCysB. Although the values of Cohen’s d, Hedges’ g, and Glass’s Delta remained high, an increase in confidence intervals was observed, reflecting greater expected population variability (Figure 7).

Together, these results indicate that both biomarkers present effect sizes compatible with clinical relevance. However, while sCysC stands out for its statistical stability and consistency across models, uCysB, despite exhibiting effects of large magnitude, proves to be more sensitive to population and methodological heterogeneity, an aspect that should be considered in the interpretation and clinical extrapolation of the findings.

### 3.4 Estimation of the ratio between groups and clinical interpretation

**Figure 8:**
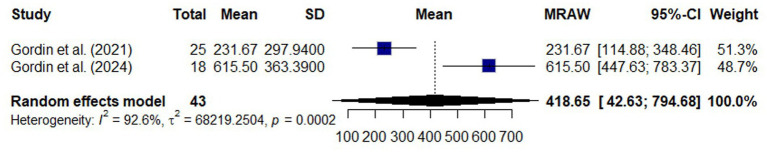
Estimated population means of urinary cystatin B (uCysB) in dogs with AKI. Weighted mean estimates indicated higher uCysB concentrations in dogs with AKI compared with healthy controls (418.65; 95% CI: 42.63–794.68). Clinically, these findings support a marked increase in uCysB during AKI, although the wide confidence interval reflects substantial variability among studies and populations.

The estimation of population means, derived from weighted means, pooled standard deviations, and standardized effect sizes, allowed inference of the expected values of the biomarkers in the control and exposed groups. For uCysB, the weighted mean in control animals was estimated at 418.65 (95% CI: 42.63 to 794.68), indicating higher values compared to controls, but with substantial variability, as reflected by the wide confidence interval. This suggests that, although the biomarker is elevated in disease, its magnitude may vary significantly depending on clinical context (Figure 8).

**Figure 9:**
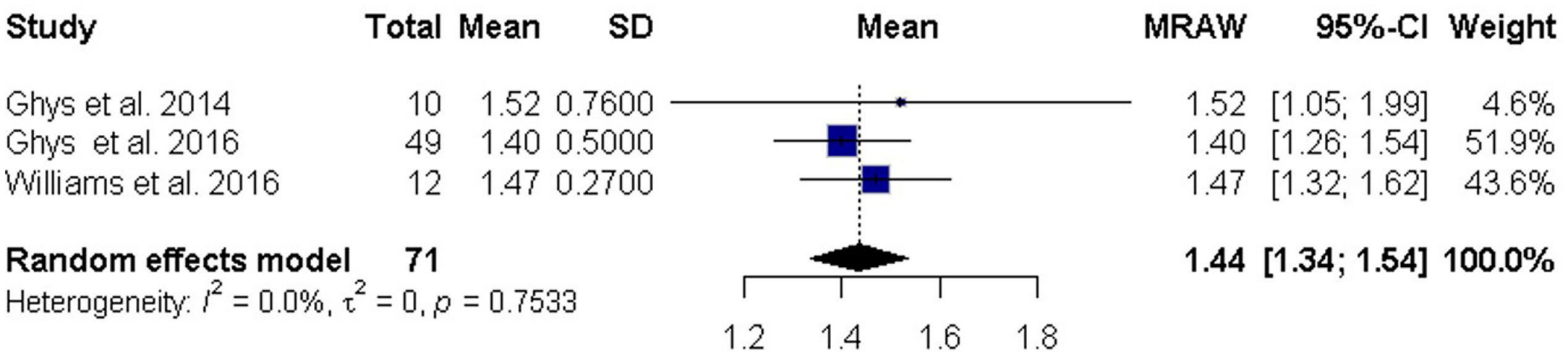
Estimated population means of sCysC in cats with CKD. The weighted mean concentration in healthy cats was 0.98 (95% CI: 0.75–1.20), while the estimated mean in cats with CKD was 1.43, corresponding to an approximately 1.47-fold increase. Clinically, these findings indicate a consistent and predictable elevation of sCysC in feline CKD.

Similarly, the analysis of sCysC, the weighted mean in control cats was 0.98 (95% CI: 0.75–1.20). Based on Glass’s Delta, the estimated mean in cats with CKD was 1.43, corresponding to an approximate 1.47-fold increase compared to controls. This estimate remained entirely within the confidence interval (95% CI: 1.34–1.54), indicating consistent and predictable elevation in diseased animals (Figure 9).

Together, these population estimates demonstrate that while uCysB presents extremely high absolute and relative increments in dogs with AKI, sCysC shows a more moderate and consistent increase in cats with CKD, reflecting important differences in the biological behavior and clinical potential of these biomarkers in distinct contexts of kidney injury. In practical terms, these magnitudes reflect a strong discriminatory capacity between healthy and diseased animals, suggesting that both sCysC and uCysB are capable of clearly separating populations with and without renal pathology at a group level (Table 3).

**Table 3:** Comparative overview of renal biomarkers used for the assessment of glomerular filtration and tubular injury.

Biomarkers	Clinical performance	Diagnostic value	Key limitations	Clinical availability
Creatinine	Late marker of GFR decline	Low sensitivity for early renal dysfunctin; high specificity for established reduction in GFR	Strongly influenced by muscle mass, diet, age, sex, and hydration status. Increases only after substantial loss of renal function (~ > 60–75% GFR reduction).	Widely available, low cost, routinely automated in clinical laboratories
SDMA	Early marker of reduced GFR	High sensitivity for mild-to-moderate decreases in GFR; moderate specificity depending on population and cutoff used	Not clearly superior to creatinine, and may yield more false positives at certain cutoffs. Biological variability in inflammatory and catabolic states.	Commercially available in veterinary diagnostic platforms; moderate cost acceptable precision, and minimal interference, enabling high-throughput clinical use
sCysC	Endogenous estimator of GFR	Good correlation with measured GFR in human medicine; moderate-to-high diagnostic performance in veterinary studies with variability	Influenced by inflammation, thyroid dysfunction, and corticosteroid therapy. Lack of full standardization across veterinary species.	Limited availability; higher cost; specialized laboratories
uCysB	Experimental biomarker of tubular injury	Insufficient evidence for pooled estimates of sensitivity or specificity. Preliminary studies suggest potential association with early tubular damage	High inter-study heterogeneity and small sample sizes. Lack of established reference intervals and validation in veterinary medicine.	uCysB is currently under clinical evaluation in veterinary medicine, although it is already used in clinical routine in some centers in the USA.

The table summarizes clinical performance, diagnostic value based on current evidence, key limitations, and clinical availability of SDMA, sCysC, and uCysB. While creatinine and SDMA are established markers of GFR, sCysC shows variable performance depending on species and clinical context. uCysB remains an experimental biomarker with limited and heterogeneous evidence, precluding definitive estimates of diagnostic accuracy and routine clinical application.

### 3.5 Statistical power

**Figure 10:**
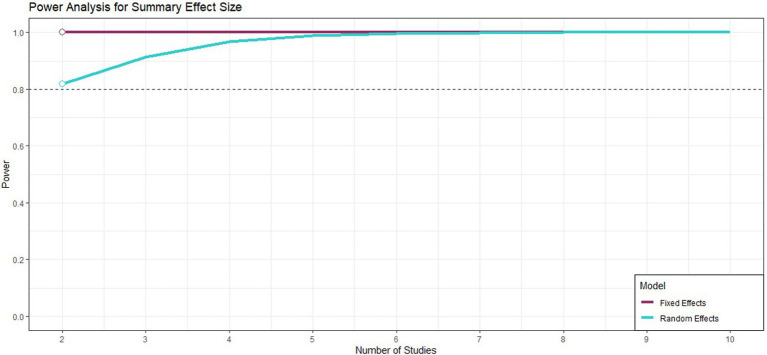
Statistical power assessment of uCysB in dogs with AKI. Power analysis based on standardized effect sizes demonstrated values close to 90%, even with only two included studies.

The assessment of statistical power, based primarily on Glass’s Delta, demonstrated values close to 90%, even with the small number of included studies. This result indicates a high probability that the observed effects represent real phenomena in the population, reducing the chance of type II errors and conferring robustness to the obtained estimates. In the case of uCysB, such statistical performance was observed even with only two studies, suggesting that, although additional investigations are desirable to refine estimates and reduce uncertainties, new studies are not strictly necessary to confirm the association with dogs AKI (Figure 10).

**Figure 11:**
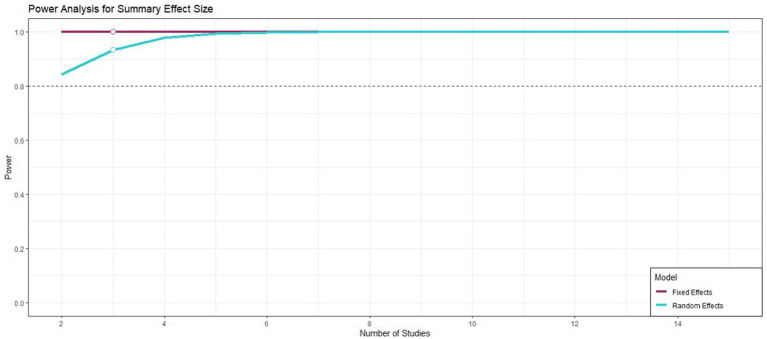
Statistical power assessment of sCysC in cats with CKD. Power analysis based on standardized effect sizes demonstrated values close to 90%, despite the limited number of included studies.

Consistent results were equally observed for sCysC, whose statistical power assessment, also derived from Glass’s Delta, indicated power close to 90%, reinforcing the statistical validity and clinical relevance of the biomarker in discriminating cats with chronic kidney disease. Complementarily, the agreement of the findings with those obtained from Cohen’s d reinforces the robustness of the results, demonstrating that the discriminatory capacity of uCysB and sCysC remains consistent regardless of the effect size metric used (Figure 11).

### 3.6 Urinary cystatin B in feline medicine: qualitative synthesis

In the present systematic review, the available evidence on uCysB in feline medicine was limited to a single study that met the predefined inclusion criteria. Due to this, a quantitative synthesis across outcomes was not feasible, and the findings were therefore summarized qualitatively.

Overall, findings from Chen et al. (18) demonstrated a progressive increase in median uCysB concentrations according to the severity of renal dysfunction, with values of 22 ng/mL in healthy cats, 171 ng/mL in UO, 112 ng/mL in CKD, and 1,052 ng/mL in AKI, with statistically significant differences among groups (*p* < 0.001). These results support a consistent pattern of uCysB elevation associated with renal injury, particularly in acute conditions.

In the association analysis, uCysB showed a moderate correlation with serum creatinine (*r* = 0.60; *p* < 0.001) and SDMA (*r* = 0.67; *p* < 0.001) in the overall population, reinforcing its relationship with conventional renal function biomarkers. However, these correlations were not maintained within the AKI subgroup alone, suggesting clinical and pathophysiological heterogeneity among different presentations of acute kidney injury.

Regarding diagnostic performance, uCysB demonstrated high accuracy for distinguishing AKI from healthy controls, with an area under the ROC curve (AUC) of 0.92 (95% CI: 0.84–1.00), sensitivity of 90%, and specificity of 92% at a cutoff value of 84 ng/mL. When the entire cohort was analyzed, diagnostic accuracy remained moderate to high (AUC = 0.83), although with the expected reduction in performance in more heterogeneous clinical settings. Additionally, uCysB showed relevant prognostic value for 30-day mortality in cats with AKI, with an AUC of 0.77 and significantly higher levels observed in non-survivors.

## 4 Discussion

This systematic review and meta-analysis evaluated the performance of sCysC and B uCysB as biomarkers of kidney disease in cats and dogs. The main findings were: (1) sCysC was elevated in cats with CKD, demonstrating large effect sizes and relatively low heterogeneity; (2) uCysB was markedly increased in dogs with AKI, exhibiting very large effect sizes despite substantial between-study heterogeneity; and (3) both biomarkers demonstrated high statistical power, supporting the biological relevance of the observed associations. Collectively, these findings reinforce the potential role of cystatins as complementary biomarkers in veterinary nephrology while highlighting important differences in the current level of evidence available for each marker.

uCysB demonstrated marked elevations in dogs with AKI compared with healthy controls, with effect sizes ranging from large to very large. This finding is consistent with the pathophysiology of AKI, in which structural tubular injury frequently precedes measurable declines in renal function (5, 6, 20). uCysB is an intracellular protein involved in the regulation of proteolytic and apoptotic pathways, and its urinary excretion is believed to reflect active cellular injury within the kidney (21). Consequently, uCysB presents an important conceptual advantage over traditional functional biomarkers, as it may identify ongoing tubular injury during a potentially reversible stage of disease (18, 20, 22, 23, 40).

The available evidence also suggests that uCysB may increase before traditional renal biomarkers such as creatinine and SDMA, supporting its potential utility for the early detection of tubular injury and subclinical renal dysfunction (19, 24). Furthermore, dogs with progressive stage 1 CKD demonstrated significantly higher uCysB concentrations than both stable CKD and control dogs, whereas no significant differences were observed for SDMA, systolic blood pressure, or urine specific gravity (20). These findings suggest that uCysB may identify ongoing renal injury before conventional markers detect functional deterioration (59, 61).

Although quantitative evidence for uCysB was predominantly derived from canine studies, the only eligible feline study also suggested potential diagnostic and prognostic relevance of this biomarker in cats with AKI. Higher uCysB concentrations were associated with poorer clinical outcomes; however, most evaluated cats already exhibited elevated creatinine concentrations at the time of diagnosis, indicating that the biomarker was assessed primarily during established stages of renal dysfunction. Therefore, although increased uCysB concentrations may reflect greater tubular injury and disease severity, the currently available feline data do not clearly demonstrate whether uCysB increases earlier than traditional functional biomarkers or provides superior diagnostic performance. Consequently, further prospective studies involving non-azotemic cats and serial monitoring throughout hospitalization are required to determine whether uCysB elevation truly precedes changes in creatinine and other conventional markers of renal function (18, 22, 23, 25).

Despite these promising findings, interpretation of uCysB requires caution. Most studies compared dogs with AKI exclusively to healthy controls, which may overestimate the discriminatory capacity of the biomarker and does not fully reflect real-world clinical decision-making. In clinical practice, differentiating AKI from other causes of azotemia is generally more relevant than distinguishing diseased animals from healthy individuals. This limitation may partially explain the substantial heterogeneity observed across studies (I^2^ = 93.7%). Variability in analytical platforms, assay calibration, AKI staging criteria, disease etiology, study populations, breed distribution, and duration of injury likely contributed to the observed dispersion of results (23, 26). Consequently, future studies should prioritize standardized assay methodologies, consensus-based AKI classification systems, and clinically relevant comparator groups to improve reproducibility and facilitate clinical translation.

Additional limitations remain regarding the biological specificity of uCysB. Correlations between urinary concentrations and renal histopathological findings are still scarce, as studies incorporating renal biopsy remain limited (27). Moreover, increased uCysB concentrations have also been reported in humans with several neoplastic conditions, potentially reflecting release from cancer cells rather than exclusively tubular injury (28–30). Therefore, although the biomarker appears promising as an indicator of active renal injury, further investigation is required to define its specificity and diagnostic positioning within broader clinical contexts.

sCysC demonstrated a distinct but equally relevant pattern. Cats with CKD consistently exhibited higher sCysC concentrations than healthy controls, with population estimates indicating an average increase of approximately 1.4–1.5-fold. The relatively narrow confidence intervals and lower heterogeneity compared with uCysB suggest a more reproducible biomarker response across studies. This finding is biologically plausible, as progressive reductions in glomerular filtration rate are expected to result in gradual accumulation of circulating sCysC (31).

One of the main theoretical advantages of sCysC is its reduced dependence on muscle mass compared with creatinine (32, 33). This characteristic has generated considerable interest in both human and veterinary nephrology, particularly for patients affected by sarcopenia or altered body composition (53). However, much of the evidence supporting this advantage originates from human medicine, where combinations of creatinine and sCysC have demonstrated utility in identifying sarcopenic individuals (34, 56, 57). In veterinary medicine, direct comparisons between sarcopenic and non-sarcopenic populations remain unavailable, and robust validation against reference methods for glomerular filtration rate, such as inulin or iohexol clearance, is still limited (54, 58).

In human medicine, sCysC has shown improved sensitivity for detecting moderate reductions in glomerular filtration rate and stronger correlations with reference GFR measurements than creatinine, especially at values close to 60 mL/min/1.73 m^2^, in addition to showing better correlation with reference methods for measuring glomerular filtration, such as radioisotopic clearance (35–38). However, comparable veterinary evidence remains scarce. Consequently, although sCysC appears to be a promising complementary marker of renal function, current data do not demonstrate clear superiority over established biomarkers such as creatinine or SDMA (48–52). At present, SDMA remains the best-validated alternative when creatinine interpretation is potentially confounded by changes in muscle mass (39, 41–47). Similar to human medicine, sCysC may currently be more appropriately viewed as an adjunctive marker of risk stratification rather than a replacement for established diagnostic tools (41, 62).

Species-specific interpretation is essential when translating these findings into clinical practice. The evidence supporting uCysB is predominantly derived from canine studies, whereas only a single feline study met the inclusion criteria of this review. Although this feline study was included qualitatively and suggested potential diagnostic and prognostic relevance, quantitative synthesis was not possible, preventing robust species-specific conclusions for cats. Conversely, the evidence regarding sCysC is primarily derived from feline CKD populations. Therefore, direct extrapolation of findings between species is not currently justified and should be approached with caution (55).

This study presents both strengths and limitations. Although the number of eligible studies was limite, statistical power approached 90% for both biomarkers, indicating a low probability of type II error and supporting the biological validity of the observed associations. Nevertheless, substantial methodological heterogeneity, differences in assay methodologies, variations in disease classification criteria, and the predominance of comparisons between diseased and healthy animals remain important limitations.

From a clinical perspective, the findings suggest that uCysB may represent a promising biomarker of active tubular injury in dogs, with potential utility for detecting renal injury before conventional functional markers become abnormal. In contrast, sCysC appears to function primarily as a complementary biomarker of CKD in cats, although current evidence does not support its replacement of creatinine or SDMA in routine clinical practice. Future research should focus on establishing standardized analytical methodologies, clinically actionable cut-off values, prognostic thresholds, and comparisons with other causes of azotemia. Such efforts will be essential to determine the true clinical value of cystatin-based biomarkers and facilitate their integration into routine veterinary nephrology.

## 5 Conclusion

sCysC and uCysB demonstrated the ability to distinguish cats and dogs with nephropathy from healthy individuals. However, sCysC in cats presented moderate elevations in CKD and, crucially, its clinical validation is limited by the scarcity of comparative studies between nephropathic animals and those with other non-renal pathologies. This methodological gap, combined with the absence of proven superiority over creatinine or SDMA, discourages its routine use in current staging and therapeutic management. On the other hand, uCysB in dogs and cats has proven to be a promising biomarker in AKI, with elevated concentrations associated with unfavorable outcomes, conferring both diagnostic and prognostic potential. Nevertheless, the implementation of this marker for early staging and personalization of therapeutic protocols in AKI still requires more robust clinical investigations.

## 6 Limitations

Despite providing a comprehensive synthesis of the available evidence, this study has limitations that should be acknowledged. The small number of eligible studies restricts species-specific conclusions and limits subgroup analyses. Substantial methodological heterogeneity was observed, including differences in assays, disease classification criteria, study populations, and AKI/CKD etiologies, which likely contributed to variability in effect estimates. In addition, most studies compared diseased animals with healthy controls, potentially overestimating diagnostic performance in real clinical settings where differentiation among disease states is required. Nevertheless, despite the limited number of included studies, statistical power analyses consistently demonstrated values close to 90% for both biomarkers, supporting that the observed associations likely represent true biological effects. These findings suggest that the currently available studies may already be sufficient to validate the existence of biomarker alterations in CKD and AKI, although additional prospective and standardized investigations remain necessary to refine prognostic utility, and clinical applicability.

## Data Availability

The original contributions presented in the study are included in the article/supplementary material, further inquiries can be directed to the corresponding author.
